# Comparative analysis of cell death induction by Taurolidine in different malignant human cancer cell lines

**DOI:** 10.1186/1756-9966-29-21

**Published:** 2010-03-07

**Authors:** Ansgar M Chromik, Adrien Daigeler, Daniel Bulut, Annegret Flier, Christina May, Kamran Harati, Jan Roschinsky, Dominique Sülberg, Peter R Ritter, Ulrich Mittelkötter, Stephan A Hahn, Waldemar Uhl

**Affiliations:** 1 Department of Visceral and General Surgery, St Josef Hospital, Ruhr-University Bochum, Germany; 2 Department of Plastic Surgery, Burn Centre, Hand Centre, Sarcoma Reference Centre, BG University Hospital Bergmannsheil GmbH, Ruhr-University Bochum, Germany; 3 Department of Medicine II, St Josef Hospital, Ruhr-University Bochum, Germany; 4 Department of Medicine I, St Josef Hospital, Ruhr-University Bochum, Germany; 5 Department of Molecular Gastrointestinal Oncology, Ruhr-University Bochum, Germany

## Abstract

**Background:**

Taurolidine (TRD) represents an anti-infective substance with anti-neoplastic activity in many malignant cell lines. So far, the knowledge about the cell death inducing mechanisms and pathways activated by TRD is limited. The aim of this study was therefore, to perform a comparative analysis of cell death induction by TRD simultaneously in different malignant cell lines.

**Materials and methods:**

Five different malignant cell lines (HT29/Colon, Chang Liver/Liver, HT1080/fibrosarcoma, AsPC-1/pancreas and BxPC-3/pancreas) were incubated with increasing concentrations of TRD (100 μM, 250 μM and 1000 μM) for 6 h and 24 h. Cell viability, apoptosis and necrosis were analyzed by FACS analysis (Propidiumiodide/AnnexinV staining). Additionally, cells were co-incubated with the caspase Inhibitor z-VAD, the radical scavenger N-Acetylcystein (NAC) and the Gluthation depleting agent BSO to examine the contribution of caspase activation and reactive oxygen species in TRD induced cell death.

**Results:**

All cell lines were susceptible to TRD induced cell death without resistance toward this anti-neoplastic agent. However, the dose response effects were varying largely between different cell lines. The effect of NAC and BSO co-treatment were highly different among cell lines - suggesting a cell line specific involvement of ROS in TRD induced cell death. Furthermore, impact of z-VAD mediated inhibition of caspases was differing strongly among the cell lines.

**Conclusion:**

This is the first study providing a simultaneous evaluation of the anti-neoplastic action of TRD across several malignant cell lines. The involvement of ROS and caspase activation was highly variable among the five cell lines, although all were susceptible to TRD induced cell death. Our results indicate, that TRD is likely to provide multifaceted cell death mechanisms leading to a cell line specific diversity.

## Background

Taurolidine (TRD), a substance derived from the aminosulfoacid Taurin, was originally used in peritonitis and catheter related blood stream infections due to its anti-microbial and anti-inflammatory properties [1-3]. Over the last years, TRD has also been shown to exert anti-neoplastic activity *in vitro *as well as *in vivo *[4]. TRD induces cell death in a variety of malignant cell lines derived from colon carcinoma [5,6], squamous cell esophageal carcinoma [7] glioblastoma [8,9], melanoma [10,11], mesothelioma [12,13] and sarcoma [14,15]. Furthermore, first reports about systemic application of TRD in patients with gastric carcinoma and glioblastoma revealed promising results with almost absent toxicity [16-18]. Favorable pharmacokinetics and safety profile of TRD render this compound to a promising agent in oncology [19].

However, mechanisms underlying induction of cell death by TRD are not yet fully elucidated. Among different types of programmed cell death (PCD) [20,21], the *classical *apoptotic cell death has been described for TRD including the *intrinsic *mitochondrial [9,12,22-24] as well as the *extrinsic *death receptor associated pathway [6,7,14,24-26]. Furthermore, there seems to be a dose dependency regarding the relative contribution to apoptotic and necrotic cell death [6,7,9,26,27]. There is an ongoing discussion about the involvement of caspase activity to TRD induced PCD. Some studies revealed enhanced caspase activity or even reversibility of TRD induced cell death by caspase-inhibition [12,13,15,22,28] whereas other denied any relevant contribution to TRD induced PCD [9,24]. As a result, additional caspase independent forms of PCD have been suggested like *autophagy *or *necrosis *[9]. Furthermore, there is growing evidence from recent publications, that generation of reactive oxygen species (ROS) plays an important role in TRD induced PCD [9,13,24,29]. However, the majority of information about TRD effects is provided from studies with one single cell line or several cell lines of one single malignancy. Methodical diversity often makes it difficult to compare results from individual cell lines and experiments. There is a lack of a comprehensive and comparative view across several cell lines of different malignancies. Furthermore, no human pancreatic cancer cell line has been evaluated for taurolidine susceptibility so far. The aim of this study was therefore, to perform a comparative analysis of cell death induction by TRD simultaneously in several cell lines of different malignancies including pancreatic cancer - focussing on dose dependency and relative contribution of apoptosis and necrosis to TRD induced cell death. Furthermore, the role of caspase activity and ROS were assessed functionally by applying specific inhibitors.

## Materials and methods

### Cell lines and culture conditions

Five different human neoplastic cancer cell lines were used for this experiment: HT29 colon carcinoma (CLS Cell Lines Service, Eppelheim, Germany), Chang Liver (HeLa contaminant, CLS Cell Lines Service, Eppelheim, Germany), HT1080 fibrosarcoma (ATCC - LGC Standards GmbH, Wesel, Germany), AsPC-1 pancreas carcinoma (CLS Cell Lines Service, Eppelheim, Germany) and BxPC-3 pancreas carcinoma (ATCC - LGC Standards GmbH, Wesel, Germany). Chang Liver cells were maintained with Dulbecco's Modified Eagle Medium (DMEM) - Hams's F12, whereas HT1080 cells were cultured in modified Eagle's medium (MEM). The remaining cell lines (HT29, AsPC-1, BxPC-3) were maintained in RPMI 1640 (Biowest, Nuaille, France). All cultures were supplemented with 10% fetal bovine serum, supplemented with penicillin (100 U/ml), streptomycin (100 μg/ml) and 2 mM L-Glutamine (Biowest, Nuaille, France). AsPC-1 and HT1080 cells were further supplemented with 1 mM Sodium Pyruvate. Cells were grown as subconfluent monolayer and cultured in 25 cm^2 ^flasks at 37°C and 5% CO_2 _in a humidified atmosphere.

### Reagents

TRD (Taurolin^®^) ultrapure powder (kindly provided by Geistlich Pharma AG, Wolhusen, Switzerland) was dissolved in a 5% Povidon solution (K16 Povidon, generously provided by Geistlich Pharma AG, Wolhusen, Switzerland) and sterile filtered to achieve the respective TRD concentrations. A 5% Povidon solution in equal volume served as a control for TRD treatment. Recombinant human TRAIL (Bender MedSystems, Vienna, Austria) was dissolved in distilled water according to the manufacturer's instructions. N-acetylcysteine (NAC) (Sigma-Aldrich, Munich, Germany) and DL-buthionin-(S,R)-sulfoximine (BSO) (Sigma-Aldrich, Munich, Germany) were dissolved in distilled water according to the manufacturer's instructions. The Caspase Inhibitor z-VAD-FMK (z-VAD) (Alexis Biochemicals, Enzo Live Sciences, Lörrach, Germany) was applied according to the manufacturer's instructions.

### Dose-effect relationship of TRD

Cells were seeded to a density of 3 × 10^6 ^cells/well in 6-well plates (growth area 9.6 cm^2^/well) and incubated for 18-24 hours under the above mentioned culture conditions to obtain a subconfluent monolayer. Subsequently, cells were washed and incubated for another 2 hours before reagents were added to the culture medium. To examine the dose-effect relationship of TRD in different malignant cell lines, cells were incubated with increasing concentrations of TRD (100, 250, and 1000 μM) and 5% Povidon as control for 6 h and 24 h. All experiments were repeated with at least 4 consecutive passages.

### Flow Cytometry Analysis and cell morphology

At the indicated incubation time, floating cells were collected together with the supernatant and adherent cells were harvested by trypsinization. Cells were sedimented by centrifugation, resuspended and fixed in 195 μl binding buffer (Bender MedSystems, Vienna, Austria). Cell density in the cell suspension was adjusted to 2 × 10^3 ^cells/μl. Subsequently, 5 μl Annexin V-FITC (BD Biosciences, Heidelberg, Germany) was added to the cell suspension followed by gently vortexing and incubation for 10 min at room temperature in the dark. Thereafter, the cell suspension was centrifuged followed by resuspension in 190 μl binding buffer before 10 μl Propidiumiodide (Bender MedSystems, Vienna, Austria) was added.

Cells were analyzed immediately using a FACS (fluoresence activated cell sorting) flow cytometer (FACS Calibur BD Biosciences, Heidelberg, Germany) for Annexin V-FITC and Propidiumiodide binding. For each measurement, 20.000 cells were counted. Dot plots and histograms were analyzed by CellQuest Pro software (BD Biosciences, Heidelberg, Germany). Annexin V positive cells were considered apoptotic; Annexin V and PI positive cells were identified as necrotic. Annexin V and PI negative cells were termed viable. Morphology of adherent cells and cells suspended in culture medium was studied and documented using a phase contrast microscope, Zeiss Axiovert 25 (Karl Zeiss, Jena, Germany). Each image was acquired at a magnification of × 20 with a spot digital camera from Zeiss.

### Contribution of reactive oxygen species to TRD induced cell death

To evaluate the contribution of reactive oxygen species (ROS) to TRD induced cell death, cells were co-incubated with TRD together with either the radical scavenger N-acetylcysteine (NAC) (5 mM) or the glutathione depleting agent DL-buthionin-(S,R)-sulfoximine (BSO) (1 mM). BSO is a selective and irreversible inhibitor of γ-glutamylcysteine synthase representing the rate-limiting biosynthetic step in glutathion snyhtesis [30,31]. In HT29, Chang Liver, HT1080 and BxPC-3 cells, TRD concentration for co-incubation was 250 μM, since there was a significant reduction of viable cells and a significant apoptotic effect in these cell lines after incubation with 250 μM as a single agent. In AsPC-1 cells, 1000 μM TRD was selected representing the only TRD dose with significant cell death induction in this particular cell line. After 6 h and 24 h, cells were analyzed by FACS for Annexin V and PI to define the relative contribution of apoptotic and necrotic cell death as described above. Results from co-incubation experiments were compared with untreated controls (Povidon 5%) and the respective single substances (TRD, NAC or BSO). Protection was considered as 'complete' when co-incubation with either NAC or BSO completely abrogated the TRD induced reduction of viable cells leading to a cell viability which was not significantly different from untreated controls. By contrast, protection was considered as 'partial' when co-incubation with either NAC or BSO decreased significantly the TRD induced reduction of viable cells without reaching the cell viability of untreated controls.

### Reversibility of TRD induced cell death by caspase inhibition

To determine the contribution of caspase activity to TRD induced cell death, cells were co-incubated with TRD (1000 μM for AsPC-1 and 250 μM HT29, Chang Liver, HT1080 and BxPC-3) and the pan-caspase inhibitor z-VAD-fmk (2 μM) for 24 h and analyzed by FACS analysis. As positive control, cells were also co-incubated with TRAIL, a known inductor of caspase dependent cell death, together with z-VAD.

### Statistical analysis

Results of FACS-analysis for percentage of viable, apoptotic and necrotic cells are expressed as means ± SEM of at least four independent experiments with consecutive passages. Comparison between experimental groups was performed using one-way ANOVA with Tukey's post-hoc text. P-values ≤ 0.05 were considered as statistically significant and indicated in the figures as follows: *** p ≤ 0.001, ** p ≤ 0.01, * p ≤ 0.05.

## Results

### TRD induces cell death in all cell lines

**Figure 1:**
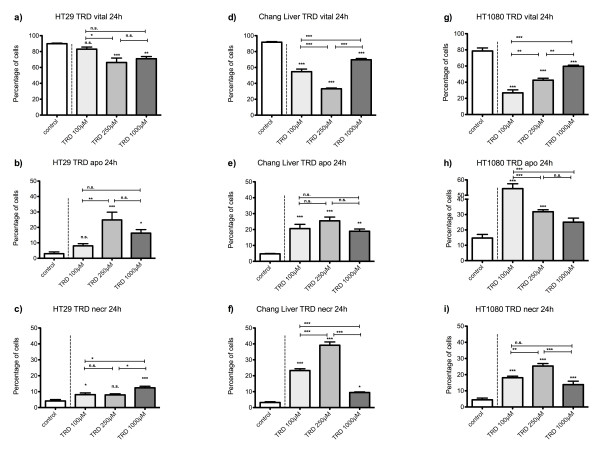
**Effects of Taurolidine on viability, apoptosis and necrosis in HT29, Chang Liver and HT1080 cells**. HT29 (a-c), Chang Liver (d-f) and HT1080 cells (g-i) were incubated with Taurolidine (TRD) (100 μM, 250 μM and 1000 μM) and with Povidon 5% (control) for 24 h. The percentages of viable (a, d, g), apoptotic (b, e, h) and necrotic cells (c, f, i) were determined by FACS-analysis for Annexin V-FITC and Propidiumiodide. Values are means ± SEM of 5 (HT29), 4 (Chang Liver) and 9 (HT1080) independent experiments with consecutive passages. Asterisk symbols on columns indicate differences between control and TRD treatment. Asterisk symbols on brackets indicate differences between TRD groups. *** p ≤ 0.001, ** p ≤ 0.01, * p ≤ 0.05 (one-way ANOVA).

**Figure 2:**
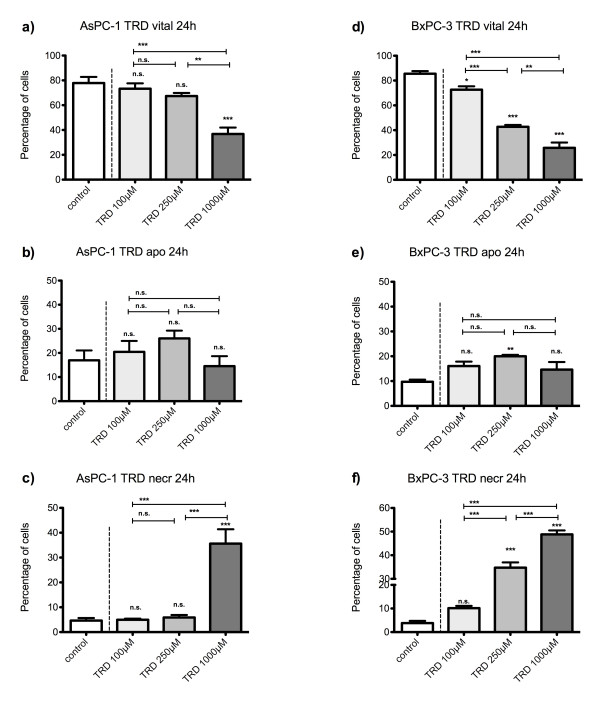
**Effects of Taurolidine on viability, apoptosis and necrosis in AsPC-1 and BxPC-3 cells**. AsPC-1 (a-c) and BxPC-3 cells (d-f) were incubated with Taurolidine (TRD) (100 μM, 250 μM and 1000 μM) and with Povidon 5% (control) for 24 h. The percentages of viable (a, d), apoptotic (b, d) and necrotic cells (c, f) were determined by FACS-analysis for Annexin V-FITC and Propidiumiodide. Values are means ± SEM of 4 independent experiments with consecutive passages. Asterisk symbols on columns indicate differences between control and TRD treatment. Asterisk symbols on brackets indicate differences between TRD groups. *** p ≤ 0.001, ** p ≤ 0.01, * p ≤ 0.05 (one-way ANOVA).

FACS analysis for Annexin V-FITC and Propidiumiodide revealed that treatment with TRD resulted in a significant reduction of viable cells compared to control treatment with Povidon 5% as early as 6 h incubation and more pronounced after 24 h (fig. 1, fig. 2, additional file 1).

### TRD induced cell death is characterized by a cell line specific contribution of apoptosis and necrosis

After 24 hours incubation, FACS analysis revealed an inhomogeneous and complex dose response effect among cell lines. In HT29 and Chang Liver cells, maximal effects on cell viability were achieved by treatment with 250 μM TRD leading to 66.2% ± 5.6% and 33.2% ± 1.0% viable cells in HT29 (fig. 1a) and Chang Liver cells (fig. 1d), respectively. In HT29 cells, this effect was due to a significant rise in apoptotic cells (fig. 1b), whereas Chang liver cells responded with significant increase in both apoptotic and necrotic cells (fig. 1e+f). In HT1080 fibrosarcoma cells, the strongest reduction of cell viability was observed after 100 μM TRD leading to 26.8% ± 3.7% viable cells (fig. 1g), mainly due to a pronounced apoptotic effect (fig. 1h). In contrast, both pancreatic cancer cell lines, AsPC-1 and BxPC-3, showed the highest response after 24 h upon treatment with 1000 μM TRD, resulting in 36.8% ± 5.2% (AsPC-1, fig. 2a) and 25.7% ± 4.3% (BxPC-3, fig. 2d) viable cells. Interestingly, this reduction of cell viability was reflected by an exclusive enhancement of necrosis without any significant effect on apoptosis. The observed proportions of necrotic cells for AsPC-1 and BxPC-3 were the highest observed in this study (fig. 2c+f) (table 1). The results for 6 hours incubation are provided in additional file 1 and summarized in table 1.

**Table 1:** Effect of increasing Taurolidine concentrations on viable, apoptotic and necrotic cells in different cell lines.

	HT29	Chang Liver	HT1080	AsPC-1	BxPC-3
**FACS analysis**					

Reduction of viable cells after 6 h	**TRD 250**	**TRD 1000**	**TRD 1000** TRD 100	**TRD 1000**	**TRD 1000** TRD 250

Increase of apoptotic cells after 6 h	**TRD 250**	**TRD 1000** TRD 250	**TRD 1000** TRD 100	**TRD 1000**	**TRD 1000** TRD 250

Increase of necrotic cells after 6 h	Ø	**TRD 1000**	**TRD 1000**	**TRD 1000**	**TRD 1000**

Reduction of viable cells after 24 h	**TRD 250** TRD 1000	**TRD 250** TRD 100 TRD 1000	**TRD 100** TRD 250 TRD 1000	**TRD 1000**	**TRD 1000** TRD 250 TRD 100

Increase of apoptotic cells after 24 h	**TRD 250** TRD 1000	**TRD 250** TRD 100 TRD 1000	**TRD 100** TRD 250 TRD 1000	Ø	TRD 250

Increase of necrotic cells after 24 h	TRD 1000	**TRD 250** TRD 100 TRD 1000	TRD 250 **TRD 100** TRD 1000	**TRD 1000**	**TRD 1000** TRD 250

Pattern of dose response (viable cells) after 24 h (FACS anaylsis)	V-shaped	V-Shaped	Anti-Prop.	Prop.	Prop.

Effect of increasing Taurolidin (TRD) concentrations (100 μM, 250 μM and 1000 μM) in different cell lines measured by FACS analysis (Annexin V/Propidium Iodide). TRD concentrations in μM with significant differences in viable, apoptotic or necrotic cells compared to untreated controls.

TRD = Taurolidin, Prop. = proportional, Anti-Prop. = anti-proportional

Ø = no significant effect

**Bold print **= TRD concentration (in μM) with the highest reduction of viable cells after 6 h and 24 h.

### TRD shows specific patterns of dose response effects among different cell lines

**Figure 3:**
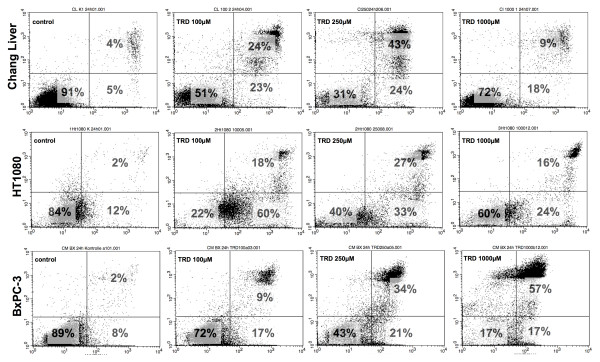
**Representative dot plots obtained by FACS-anaylsis after incubation of different cell lines with Taurolidine**. Chang Liver, HT1080 and BxPC-3 cells were incubated with Taurolidine (TRD) (100 μM, 250 μM and 1000 μM) and with Povidon 5% (control) for 24 h. FACS-analysis was performed for Annexin V-FITC (x-axis) and Propidiumiodide (y-axis). Lower left quadrant: Annexin V and propidium iodide negative (viable), lower right quadrant: Annexin V positive and propidium iodide negative (apoptotic), upper right quadrant: Annexin V and propidium iodide positive (necrotic).

Dose response effects after 24 h were neither straight proportional nor uniform among different cell lines. The only cell line with an obvious proportional dose effect was BxPC-3. In this cell line, all TRD concentrations (100 μM, 250 μM and 1000 μM) caused a significant reduction of viable cells compared to control treatment with significant differences between increasing TRD concentrations (fig. 2d). The other pancreatic cancer cell line, AsPC-1, displayed at least some characteristics of a proportional dose effect. The reduction of viable cells with increasing TRD concentrations became statistically significant for 1000 μM TRD, as illustrated in fig. 2a. Two cell lines were characterized by an V-shaped dose response pattern after 24 h. HT29 and Chang Liver cells had the maximal reduction of viable cells after incubation with 250 μM TRD, which represents the intermediate concentration between 100 μM and 1000 μM TRD (fig. 1a+d). Unlike all other cell lines, HT1080 cells demonstrated an anti-proportional dose response with the highest reduction of viable cells by 100 μM TRD. Both following concentrations - 250 μM and 1000 μM TRD - were also capable of a significant reduction of cell viability - but not as strongly as 100 μM TRD (fig.1g) (table 1). Representative FACS dot plots for Chang Liver, HT1080 and BxPC-3 cells are presented in figure 3 - indicating the different patterns of dose response among these cell lines (fig. 3).

### The radical scavenger N-acetylcysteine (NAC) and the glutathione depleting agent L-S, R-Buthionine sulfoximine (BSO) show cell line specific and divergent effects on TRD induced cell death

**Figure 4:**
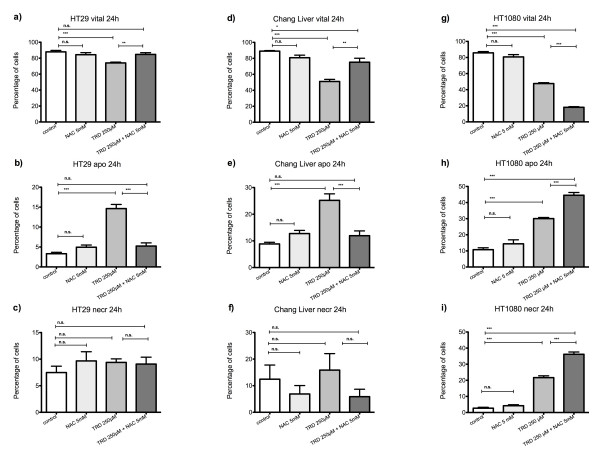
**Effects of N-acetylcysteine on Taurolidine induced cell death in HT29, Chang Liver and HT1080 cells**. HT29 (a-c), Chang Liver (d-f) and HT1080 cells (g-i) were incubated with either the radical scavenger N-acetylcysteine (NAC) (5 mM), Taurolidine (TRD) (250 μM) or the combination of both agents (TRD 250 μM + NAC 5 mM) and with Povidon 5% (control) for 24 h. The percentages of viable (a, d, g), apoptotic (b, e, h) and necrotic cells (c, f, i) were determined by FACS-analysis for Annexin V-FITC and Propidiumiodide. Values are means ± SEM of 4 (HT29 and Chang Liver) and 12 (HT1080) independent experiments with consecutive passages. Asterisk symbols on brackets indicate differences between treatment groups. *** p ≤ 0.001, ** p ≤ 0.01, * p ≤ 0.05 (one-way ANOVA).

**Figure 5:**
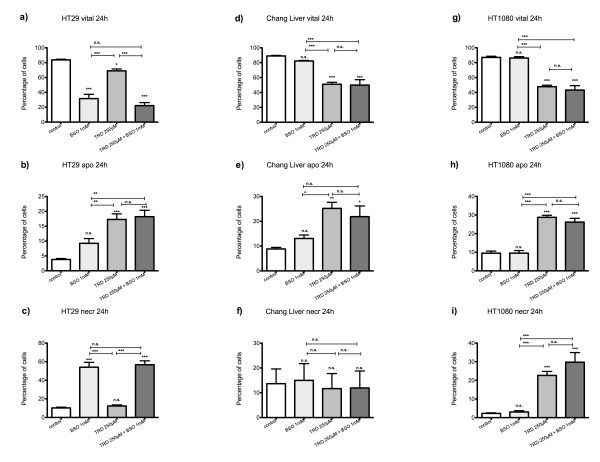
**Effects of DL-buthionin-(S,R)-sulfoximine on Taurolidine induced cell death in HT29, Chang Liver and HT1080 cells**. HT29 (a-c), Chang Liver (d-f) and HT1080 cells (g-i) were incubated with either the glutathione depleting agent DL-buthionin-(S,R)-sulfoximine(BSO) (1 mM), Taurolidine (TRD) (250 μM) or the combination of both agents (TRD 250 μM + BSO 1 mM) and with Povidon 5% (control) for 24 h. The percentages of viable (a, d, g), apoptotic (b, e, h) and necrotic cells (c, f, i) were determined by FACS-analysis for Annexin V-FITC and Propidiumiodide. Values are means ± SEM of 9 (HT29 and HT1080) and 4 (Chang Liver) independent experiments with consecutive passages. Asterisk symbols on brackets indicate differences between treatment groups. *** p ≤ 0.001, ** p ≤ 0.01, * p ≤ 0.05 (one-way ANOVA).

In HT29 colon carcinoma cells, co-incubation of TRD with NAC for 24 h led to a complete protection of TRD induced cell death. NAC completely abrogated the TRD induced reduction of viable cells leading to a cell viability which was not different from untreated controls (fig. 4a). This effect was related to a significant reduction of apoptotic cells compared to TRD alone (fig. 4b). Consistent with this finding, co-incubation with the glutathione depleting compound BSO for 24 h led to a significant enhancement of TRD induced cell death which was caused by a significant increase in necrosis (fig. 5a+c) (table 2). However, BSO itself also reduced cell viability significantly through pronounced necrosis (fig. 5a+c) (table 2).

**Table 2:** Effect of N-Acetylcystein, DL-buthionin-(S,R)-sulfoximine or z-VAD co-incubation with Taurolidine in different cell lines.

		HT29	Chang Liver	HT1080	AsPC-1	BxPC-3
NAC+TRD 6 h	Viable:	Ø	Ø	Ø	CoProt	Ø
	Apo/Nec:	Apo⇓	Ø	Nec⇓	Nec⇓	Ø

NAC+TRD 24 h	Viable:	CoProt	PaProt.	Del	PaProt	PaProt
	Apo/Nec:	Apo⇓	Apo⇓	Apo⇑ Nec⇑	Nec⇓ Apo⇑	Nec⇓

BSO alone 6 h	Viable:	Ø	Ø	Ø	Ø	Ø
	Apo/Nec	Ø	Ø	Ø	Ø	Ø

BSO+TRD 6 h	Viable:	Ø	Ø	Ø	Ø	Del
	Apo/Nec:	Ø	Ø	Nec⇓	Nec⇑ Apo⇓	Nec⇑

BSO alone 24 h	Viable:	Del	Ø	Ø	Ø	Del
	Apo/Nec:	Nec⇑	Ø	Ø	Ø	Nec⇑

BSO+TRD 24 h	Viable:	Del	Ø	Ø	Del	Del
	Apo/Nec:	Nec⇑	Ø	Ø	Nec⇑	Apo⇑ Nec⇓

z-VAD+ TRD 24 h	Viable:	CoProt	PaProt	PaProt	Ø	Ø
	Apo/Nec:	Apo⇓	Ø	Nec⇓	Nec⇑	Nec⇓

Effect of N-Acetylcystein (NAC), DL-buthionin-(S,R)-sulfoximine (BSO) or z-VAD co-incubation with Taurolidin (TRD) in different cell lines measured by FACS analysis (Annexin V/Propidium Iodide).

NAC = N-Acetylcysteine

BSO = DL-buthionin-(S,R)-sulfoximine

TRD = Taurolidine

Viable = viable cells

Apo = apoptotic cells

Nec = necrotit cells

Ø = no significant effect

⇓ = significant decrease

⇑ = significant increase

CoProt. = complete protection

PaProt. = partial protection

Del. = deleterious

**Figure 6:**
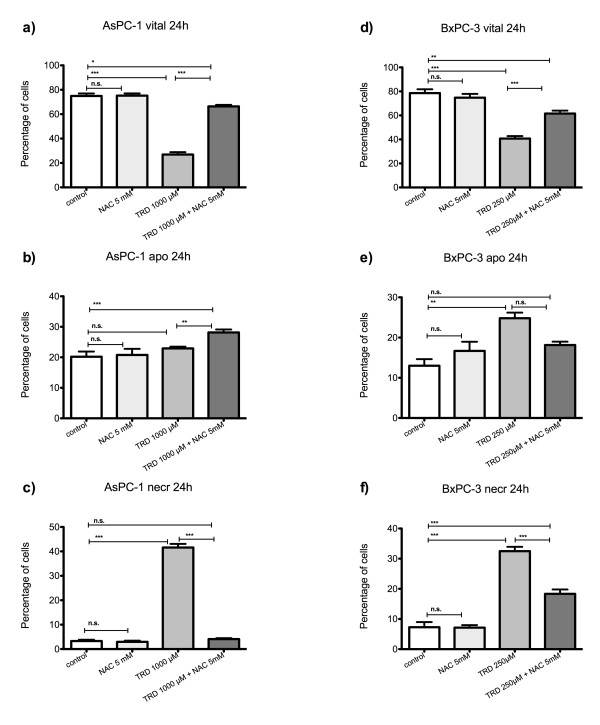
**Effects of N-acetylcysteine on Taurolidine induced cell death in AsPC-1 and BxPC-3 cells**. AsPC-1 (a-c) and BxPC-3 cells (d-f) were incubated with either the radical scavenger N-acetylcysteine (NAC) (5 mM), Taurolidine (TRD) (250 μM for BxPC-3 and 1000 μM for AsPC-1) or the combination of both agents (TRD 250 μM/1000 μM + NAC 5 mM) and with Povidon 5% (control) for 24 h. The percentages of viable (a, d), apoptotic (b, e) and necrotic cells (c, f) were determined by FACS-analysis for Annexin V-FITC and Propidiumiodide. Values are means ± SEM of 4 independent experiments with consecutive passages. Asterisk symbols on brackets indicate differences between treatment groups. *** p ≤ 0.001, ** p ≤ 0.01, * p ≤ 0.05 (one-way ANOVA).

**Figure 7:**
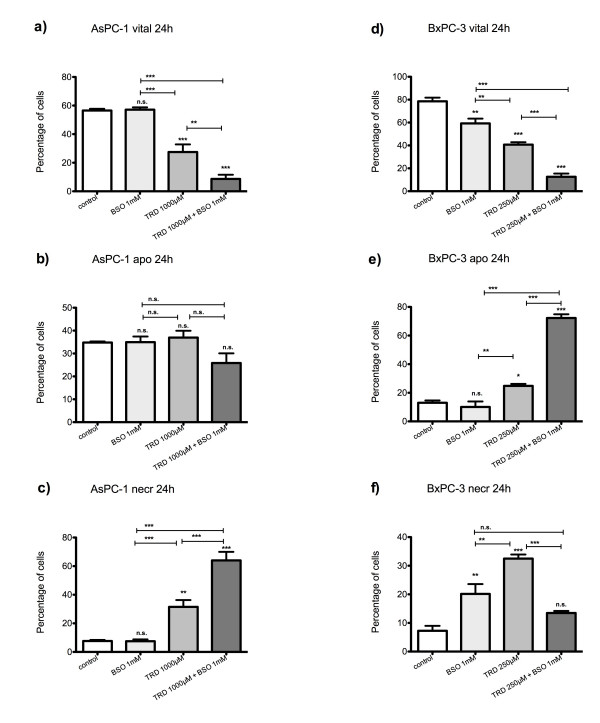
**Effects of DL-buthionin-(S,R)-sulfoximine on Taurolidine induced cell death in AsPC-1 and BxPC-3 cells**. AsPC-1 (a-c) and BxPC-3 cells (d-f) were incubated with either the glutathione depleting agent DL-buthionin-(S,R)-sulfoximine(BSO) (1 mM), Taurolidine (TRD) (250 μM for BxPC-3 and 1000 μM for AsPC-1) or the combination of both agents (TRD 250 μM/1000 μM + BSO 1 mM) and with Povidon 5% (control) for 24 h. The percentages of viable (a, d), apoptotic (b, e) and necrotic cells (c, f) were determined by FACS-analysis for Annexin V-FITC and Propidiumiodide. Values are means ± SEM of 4 independent experiments with consecutive passages. Asterisk symbols on brackets indicate differences between treatment groups. *** p ≤ 0.001, ** p ≤ 0.01, * p ≤ 0.05 (one-way ANOVA).

In AsPC-1 cells, NAC co-incubation was characterized by a strong reduction of necrosis compared to TRD alone (fig. 6c). Together with a small - but significant - increase in apoptotic cells (fig. 6b) this effect led to a significant increase in viable cells compared to TRD alone (fig. 6a). However, there was no complete recovery in the proportion of viable cells compared to untreated controls (fig. 6a). For that reason the effect could only be designated as partial protection (table 2). In line with the protective effects of NAC, co-incubation with BSO resulted in a significant increase of necrotic cells compared to TRD alone (fig. 7c) leading to a deleterious effect on cell viability after (fig. 7a). It is important to note, that BSO as a single agent had no significant effect on cell viability, apoptosis and necrosis in this particular cell line (fig. 7a-c).

The second pancreatic cancer cell line, BxPC3, showed some similarities with AsPC-1 cells regarding the response to NAC and BSO co-incubation (fig. 6+7;d-f). A partial protective effect of NAC co-incubation could be demonstrated leading to a significant increase in viable cells compared to TRD alone without full recovery compared to untreated controls (fig. 6d). This partial recovery by NAC was again related to a reduction of necrotic cells compared to TRD alone (fig. 6f) (table 2). Unlike AsPC-1 cells, BxPC-3 cells responded to BSO as a single agent with a significant reduction of viable cells compared to untreated controls (fig. 7d+f). Nevertheless, there was again a significant deleterious effect of BSO co-incubation with TRD on cell viability compared to TRD or BSO alone (fig. 7d), which was related to a strong enhancement of apoptosis (fig. 7e).

Chang Liver cells responded least to NAC and BSO co-incubation (fig. 4+5; d-f). However after 24 h, a partial protection by NAC co-incubation could be encountered leading to a significant increase in viable cells compared to TRD alone without complete recovery compared to control treatment (fig. 4d). The partial protective effect was characterized by a significant decrease in apoptotic cells compared to TRD alone (fig. 4e+f). Co-incubation with BSO did not result in any significant effect on cell viability, apoptosis and necrosis compared to TRD alone (fig. 6d-f) (table 2).

Compared to all other cell lines, HT1080 cells were characterized by a unique and occasionally completely contrary response to radical scavenging by NAC (fig. 4g-i). NAC co-incubation did not result in cell rescue but led to further significant reduction of viable cells compared to TRD alone (fig. 4g). This deleterious effect of NAC was mirrored by significantly enhanced apoptosis and necrosis compared to TRD alone (fig. 4h+i). Co-incubation with BSO did not result in any significant effect on cell viability, apoptosis and necrosis compared to TRD alone (fig. 5g-i).

The results for 6 hours co-incubation with NAC and BSO are provided in additional file 2 and 3, respectively and summarized in table 2.

### The reversibility of TRD induced cell death by caspase inhibition is divergent and cell line specific

**Figure 8:**
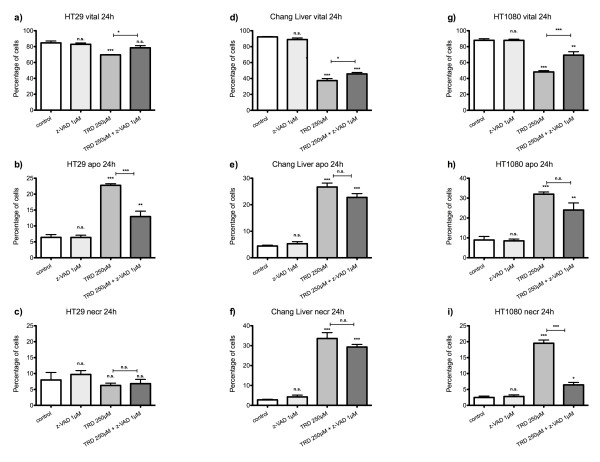
**Effects of caspase-inhibition on Taurolidine induced cell death in HT29, Chang Liver and HT1080 cells**. HT29 (a-c), Chang Liver (d-f) and HT1080 cells (g-i) were incubated with either z-VAD.fmk (1 μM), Taurolidine (TRD) (250 μM) or the combination of both agents (TRD 250 μM + zVAD.fmk 1 μM) and with Povidon 5% (control) for 24 h. The percentages of viable (a, d, g), apoptotic (b, e, h) and necrotic cells (c, f, i) were determined by FACS-analysis for Annexin V-FITC and Propidiumiodide. Values are means ± SEM of 5 (HT29), 6 (Chang Liver) and 4 (HT1080) independent experiments with consecutive passages. Asterisk symbols on brackets indicate differences between treatment groups. *** p ≤ 0.001, ** p ≤ 0.01, * p ≤ 0.05 (one-way ANOVA).

Overall, there was no effect on cell viability, apoptosis or necrosis of z-VAD alone in any of the five cell lines. HT29 was the only cell line with a complete protection of TRD induced cell death by z-VAD co-incubation and thus a complete reversibility of TRD induced cell death (fig. 8a). The relatively mild reduction of viable cells by TRD to 69.6% ± 0.3% was significantly abrogated by z-VAD co-incubation and not different from untreated controls (fig. 8a). The protective effect was associated with a significant decrease of apoptotic cells (fig. 8b) without any detectable effect on necrosis (fig. 8c).

**Figure 9:**
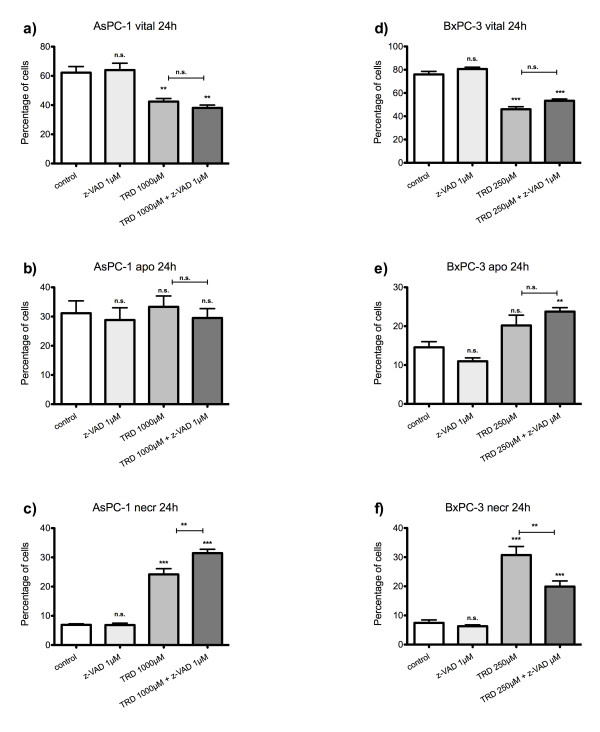
**Effects of caspase-inhibition on Taurolidine induced cell death in AsPC-1 and BxPC-3 cells**. AsPC-1 (a-c) and BxPC-3 cells (d-f) were incubated with either z-VAD.fmk (1 μM), Taurolidine (TRD) (250 μM for BxPC-3 and 1000 μM for AsPC-1) or the combination of both agents (TRD 250 μM/1000 μM + zVAD.fmk 1 μM) and with Povidon 5% (control) for 24 h. The percentages of viable (a, d), apoptotic (b, e) and necrotic cells (c, f) were determined by FACS-analysis for Annexin V-FITC and Propidiumiodide. Values are means ± SEM of 3 (AsPC-1) and 6 (BxPC-3) independent experiments with consecutive passages. Asterisk symbols on brackets indicate differences between treatment groups. *** p ≤ 0.001, ** p ≤ 0.01, * p ≤ 0.05 (one-way ANOVA).

In Chang Liver and HT1080 cells, the TRD induced cell death was only partially reversible by z-VAD dependent caspase inhibition. The rescue effect of z-VAD co-incubation did not lead to the same cell viability like untreated controls. In Chang Liver cells, the protective effect of z-VAD co-incubation compared to TRD alone was relatively small (45.7% ± 1.8% vs. 37.4% ± 2.6%) although it reached statistical significance (fig. 8d). This partial rescue effect of z-VAD was paralleled by a small and non-significant reduction of both apoptotic and necrotic cells (fig. 8e+f). HT1080 cells responded similar to z-VAD co-incubation with a partial protective effect characterized by a significantly increased cell viability compared to TRD alone but not compared to untreated (fig. 8g). The partial protection by z-VAD was mainly achieved by a significant reduction of necrosis (fig. 8i). Both pancreatic cancer cell-lines, AsPC-1 and BxPC-3 did not show any detectable effect on cell viability after z-VAD co-incubation. In AsPC-1 cells, TRD 1000 μM induced reduction of viable cells could not be reversed by z-VAD co-incubation (fig. 9a). In contrast, z-VAD co-incubation resulted in a significant increase in necrotic cells (fig. 9c). In BxPC-3 cells, the TRD induced reduction of viable cells could not significantly be reversed by z-VAD co-incubation (fig. 9d) although there was a significant decrease in necrotic cells following z-VAD co-incubation compared to TRD alone (fig. 9f) (table 2).

## Discussion

Although the anti-neoplastic effects of TRD have been extensivley analyzed *in vitro *by proliferation assays like BrdU or MTT [12-14,27,28,32], only few studies have exploited the potential of FACS analysis to differentiate in a quantitative manner between apoptotic and necrotic cell death [13,26,33,34]. Furthermore, all available studies were performed on single cell lines or on different cell lines of one particular malignancy. There is a lack of a comparative analysis of TRD effects in cell lines of different malignancies including pancreatic cancer. Therefore, in the first part of this study we sought to determine dose-response characteristics and relative contribution of apoptosis and necrosis of TRD induced cell death simultaneously in 5 cell lines from 4 malignancies. Surprisingly, dose response effects of TRD were not homogenous among the 5 cell lines. In fact, we found three different patterns of dose response: *proportional, V-shaped *and *anti-proportional dose effects*. The two pancreatic cancer cell lines BxPC-3 and AsPC-1 which have never been tested before, were characterized by a *proportional dose effect*. Increasing concentrations of TRD led to increasing cell death after 6 and 24 hours. The *proportional dose effect *pattern of TRD has been described in the majority of available studies either by proliferation assays [11-13,28,32,35], FACS analysis [7] or other cell viability/toxicity assays [9,27,36]. BxPC-3 cells displayed also a dose dependency regarding the relative contribution of necrotic and apoptotic cell death. The response on cell viability upon incubation with TRD 250 μM for 24 hours was characterized by a mixed apoptotic and necrotic effect whereas TRD 1000 μM was characterized by an exclusive and pronounced necrotic effect. This phenomenon became even more obvious in AsPC-1 cells, were TRD 1000 μM led to a strong necrotic effect. The observed dose dependency of apoptotic and necrotic cell death is consistent with previous studies by others [27] as well as by our group [6,26,34]. The *V-shaped dose effect *was found in HT29 cells as well as in Chang Liver cells and was characterized by a dose response with maximal effects on cell viability and apoptosis with the intermediated concentration of TRD 250 μM whereas the highest (TRD 1000 μM) and lowest (TRD 100 μM) concentrations were less effective. This *V-shaped dose effect *has been described only once by our group [34]. However, to our surprise HT1080 cells presented in the current study with a *anti-proportional dose effect *with decreasing effects on cell viability and apoptosis upon treatment for 24 h with increasing TRD concentrations. We can only speculate about the reason for this inverse proportionality. Our assays were repeated with nine consecutive passages, thus excluding biological assay variability as a possible explanation for this unusual finding.

The second part of the study comprised the evaluation of the contribution of reactive oxygen species (ROS) to TRD induced PCD by co-incubation experiments with either the radical scavenger N-acetylcysteine (NAC) or the glutathione depleting agent DL-buthionin-(S,R)-sulfoximine (BSO). Previous studies have presented first evidence for involvement of TRD mediated ROS production [9,13,36]. Furthermore, cell death induced by TRD has been shown to be reversible by application of radical scavengers like NAC [9,12,13,36] and to be enhanced by inhibitors of ROS detoxification like BSO [9]. In our study, all cell lines except HT1080 fibrosarcoma cells responded to NAC co-incubation with an attenuation of TRD induced cell death. However, the magnitude of protection was divergent among cell lines ranging from partial protection (Chang Liver, AsPC-1, BxPC-3) to complete protection (HT29). To our surprise and in contrast to the available literature, HT1080 cells presented a completely contrary response to radical scavenging by NAC leading to enhancement rather than attenuation of TRD induced cell death. The biological cause behind this unexpected response pattern is currently unknown. However, ROS can be regarded as a "double edged sword" in terms of anti-neoplastic activity [37]. Excessive ROS generation in tumor cells and subsequent activation of PCD is a well known therapeutic principle of many chemotherapeutics e.g. anthracyclines, platinum or arsenic [37-40]. On the other hand, ROS can promote tumor cell proliferation and survival under certain circumstances [37,41] and anti-oxidant therapeutics may provide anti-neoplastic activity by inhibiting ROS production [37]. In conclusion, generation of ROS and activation of subsequent pathways does explain TRD induced cell death in many, but obviously not in every cell line or malignancy. ROS generation is rather unlikely to be the universal key mechanism of TRD induced PCD in all cell lines.

The second major cell death associated pathway analyzed in this study was the caspase pathway by applying the pan caspase inhibitor z-VAD. Activation of the caspase pathway by TRD has been reported in several malignant cell lines [12,13,15,22]. Concordant with the divergent and cell line specific results of our ROS experiments - we encountered an inhomogeneous response to co-treatment with z-VAD among our 5 cell lines. Z-VAD was capable of protecting tumor cells from TRD induced cell death only in HT29 (complete protection), Chang Liver and HT1080 cells (partial protection), but both pancreatic cancer cell lines AsPC-1 and BxPC-3 were not protected at all. Comparable divergent findings about the contribution of caspase activity to TRD induced cell death have recently been reported by others [9,15,28,36] suggesting both caspase dependent and independent pathways [12]. During the last years, it became clear that PCD can occur independently of caspase activation which is no longer regarded as a mandatory feature of PCD [20,42,43]. Interestingly, AIF (apoptosis inducing factor) representing a key protein in caspase independent PCD has recently been shown to be involved in TRD induced cell death [9,36]. However, no study has provided a comparative analysis of caspase inhibition and TRD simultaneously in different cell lines.

The herein observed divergent response in cell lines of different malignancies towards inhibition of TRD induced cell death by z-VAD as well as by NAC leads to the assumption, that there is a cell line specificity regarding involvement of caspases and ROS following TRD treatment. Further studies are necessary to elucidate the different types of programmed cell death following TRD treatment.

## Conclusions

This is the first study providing a simultaneous evaluation of TRD induced cell death across several cell lines of different malignancies. TRD is characterized by cell line specific dose response effects and dose response patterns. However, all cell lines were susceptible to TRD induced cell death without any resistance. Functional analysis for involvement of ROS driven cell death and caspase activation revealed substantial cancer cell type specific differences for both routes of cell death. Thus, TRD is likely to provide multifaceted cell death mechanisms leading to a cell line specific diversity.

## Abbreviations

BSO: DL-buthionin-(S,R)-sulfoximine; PCD: Programmed cell death; NAC: N-Acetylcysteine; ROS: Reactive oxygen species; TRAIL: Tumor Necrosis Factor Related Apoptosis Inducing Ligand; TRD: Taurolidine

## Competing interests

AMC received financial support by Geistlich Pharma (Suisse) for laboratory experiments. All other authors declare that they have no competing interests.

## Authors' contributions

AMC and AD conceived of the study and its design, coordinated the experiments, carried out the statistical analysis and drafted the manuscript. AF supervised the cell culture experiments and carried out the inhibitor experiments. DB was responsible for adjusting the FACS analysis and helped to draft the manuscript. CM, KH and JR carried out the cell culture experiments. DS helped with the statistical analysis and revised manuscript. PR, UM, SH and WU participated in the design and coordination of the study and revised the manuscript. All authors have read and approved the final manuscript.

## Supplementary Material

Additional file 1 **Effects of Taurolidine on viability, apoptosis and necrosis in HT29, Chang Liver, HT1080, AsPC-1 and BxPC-3 cells after 6 h**. HT29, Chang Liver, HT1080, AsPC-1 and BxPC-3 cells were incubated with Taurolidine (TRD) (100 μM, 250 μM and 1000 μM) and with Povidon 5% (control) for 6 h. The percentages of viable (vital), apoptotic (apo) and necrotic cells (necr) were determined by FACS-analysis for Annexin V-FITC and Propidiumiodide. Values are means ± SEM of 5 (HT29), 4 (Chang Liver, AsPC-1 and BxPC-3) and 9 (HT1080) independent experiments with consecutive passages. Asterisk symbols on columns indicate differences between control and TRD treatment. Asterisk symbols on brackets indicate differences between TRD groups. *** p ≤ 0.001, ** p ≤ 0.01, * p ≤ 0.05 (one-way ANOVA). Click here for file

Additional file 2 **Effects of N-acetylcysteine on Taurolidine induced cell death in HT29, Chang Liver, HT1080, AsPC-1 and BxPC-3 cells after 6 h**. HT29, Chang Liver, HT1080, AsPC-1 and BxPC-3 cells were incubated with either the radical scavenger N-acetylcysteine (NAC) (5 mM), Taurolidine (TRD) (250 μM) or the combination of both agents (TRD 250 μM + NAC 5 mM) and with Povidon 5% (control) for 6 h. The percentages of viable (vital), apoptotic (apo) and necrotic cells (necr) were determined by FACS-analysis for Annexin V-FITC and Propidiumiodide. Values are means ± SEM of 4 (HT29, Chang Liver, AsPC-1 and BxPC-3) and 12 (HT1080) independent experiments with consecutive passages. Asterisk symbols on brackets indicate differences between treatment groups. *** p ≤ 0.001, ** p ≤ 0.01, * p ≤ 0.05 (one-way ANOVA). Click here for file

Additional file 3 **Effects of DL-buthionin-(S,R)-sulfoximine on Taurolidine induced cell death in HT29, Chang Liver, HT1080, AsPC-1 and BxPC-3 cells after 6 h**. HT29, Chang Liver, HT1080, AsPC-1 and BxPC-3 cells were incubated with either the glutathione depleting agent DL-buthionin-(S,R)-sulfoximine (BSO) (1 mM), Taurolidine (TRD) (250 μM) or the combination of both agents (TRD 250 μM + BSO 1 mM) and with Povidon 5% (control) for 6 h. The percentages of viable (vital), apoptotic (apo) and necrotic cells (necr) were determined by FACS-analysis for Annexin V-FITC and Propidiumiodide. Values are means ± SEM of 9 (HT29 and HT1080) and 4 (Chang Liver, AsPC-1 and BxPC-3) independent experiments with consecutive passages. Asterisk symbols on brackets indicate differences between treatment groups. *** p ≤ 0.001, ** p ≤ 0.01, * p ≤ 0.05 (one-way ANOVA). Click here for file
