# Supplementary figures and images for: Comparative analysis of cell death induction by Taurolidine in different malignant human cancer cell lines
Source: J Exp Clin Cancer Res. 2010 Mar 7;29(1):21. doi: 10.1186/1756-9966-29-21 (PMC2846881; doi:10.1186/1756-9966-29-21)

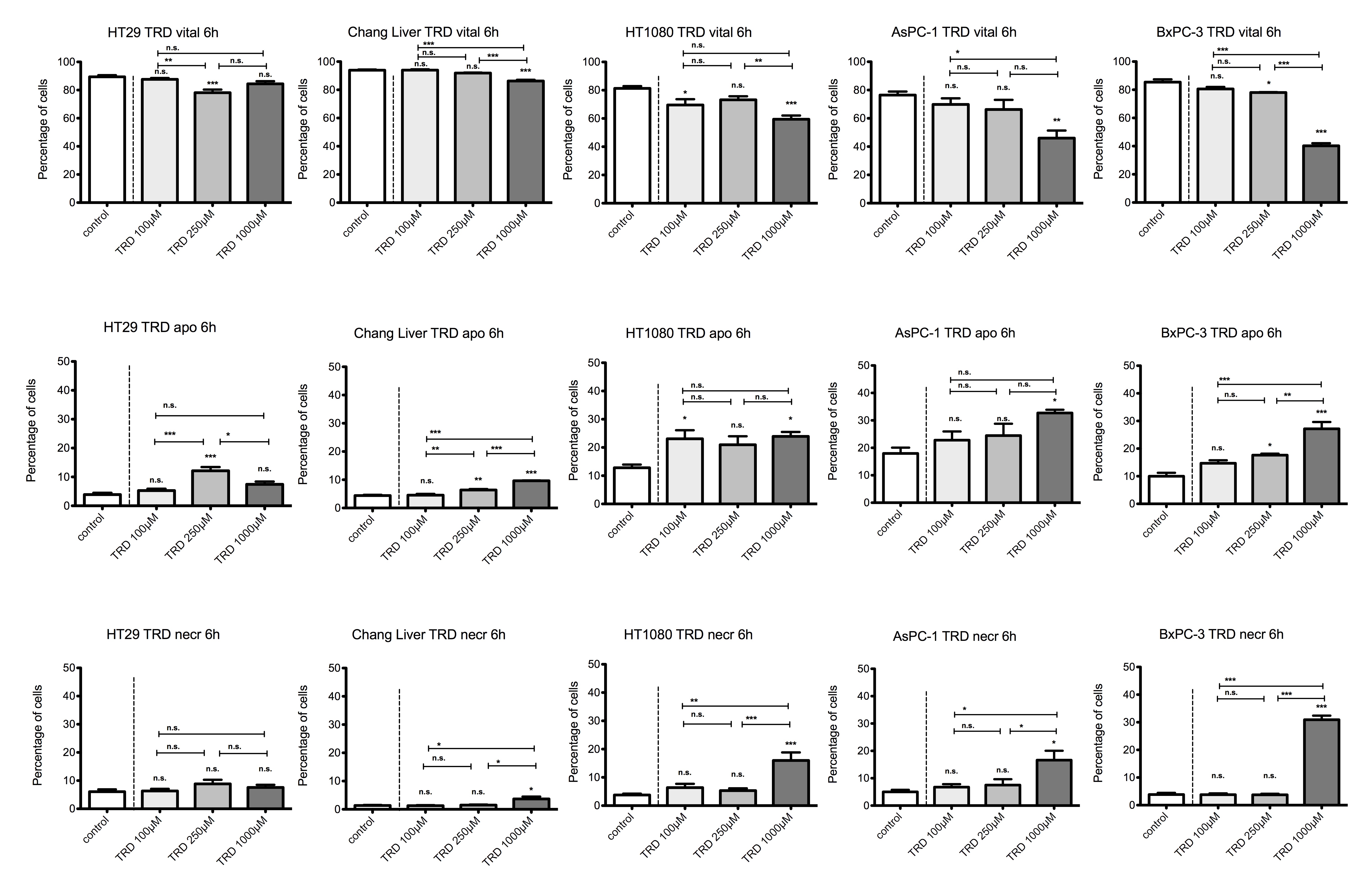

Supplement: Additional file 1 — Effects of Taurolidine on viability, apoptosis and necrosis in HT29, Chang Liver, HT1080, AsPC-1 and BxPC-3 cells after 6 h. HT29, Chang Liver, HT1080, AsPC-1 and BxPC-3 cells were incubated with Taurolidine (TRD) (100 μM, 250 μM and 1000 μM) and with Povidon 5% (control) for 6 h. The percentages of viable (vital), apoptotic (apo) and necrotic cells (necr) were determined by FACS-analysis for Annexin V-FITC and Propidiumiodide. Values are means ± SEM of 5 (HT29), 4 (Chang Liver, AsPC-1 and BxPC-3) and 9 (HT1080) independent experiments with consecutive passages. Asterisk symbols on columns indicate differences between control and TRD treatment. Asterisk symbols on brackets indicate differences between TRD groups. *** p ≤ 0.001, ** p ≤ 0.01, * p ≤ 0.05 (one-way ANOVA). [file 1756-9966-29-21-S1.JPEG]

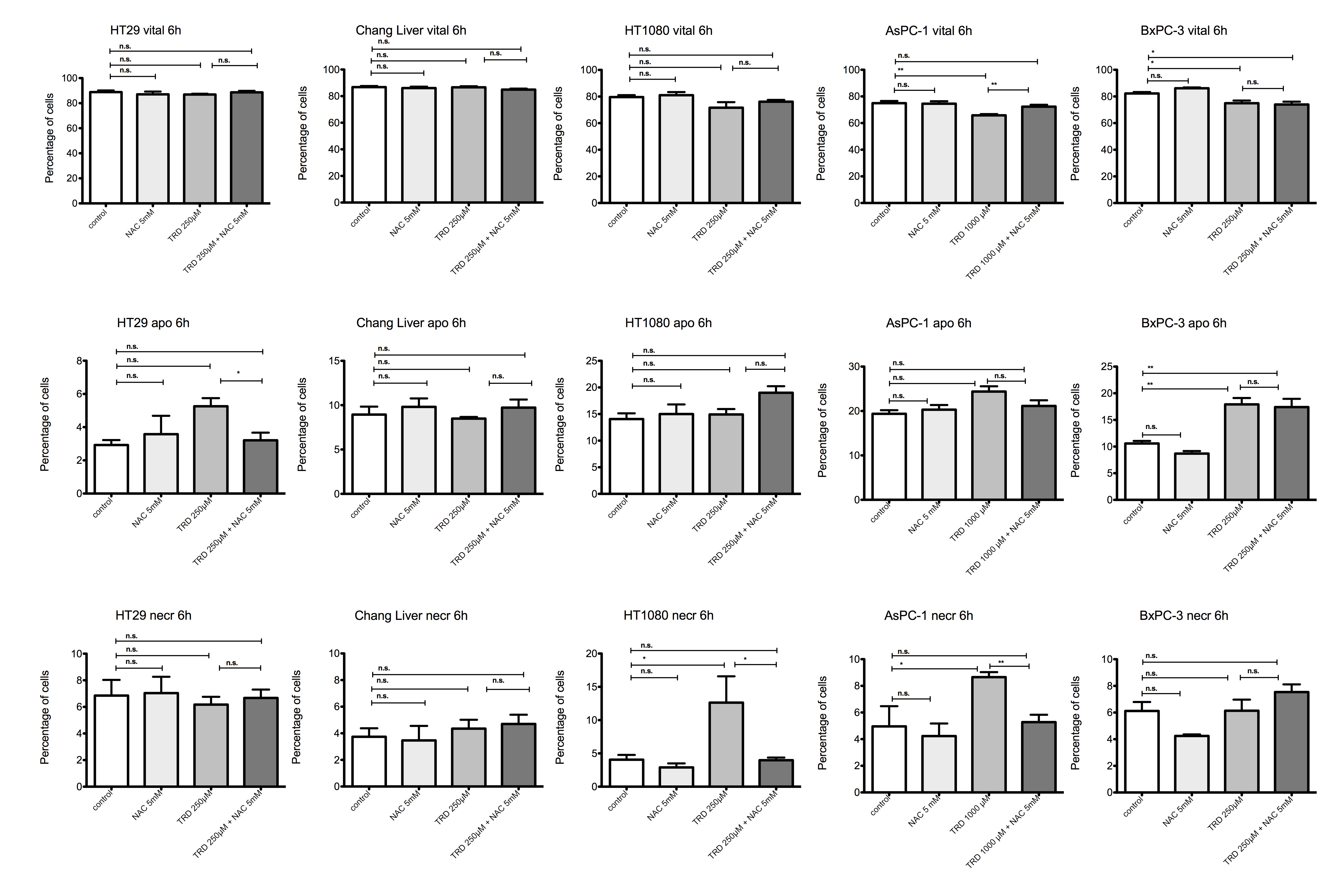

Supplement: Additional file 2 — Effects of N-acetylcysteine on Taurolidine induced cell death in HT29, Chang Liver, HT1080, AsPC-1 and BxPC-3 cells after 6 h. HT29, Chang Liver, HT1080, AsPC-1 and BxPC-3 cells were incubated with either the radical scavenger N-acetylcysteine (NAC) (5 mM), Taurolidine (TRD) (250 μM) or the combination of both agents (TRD 250 μM + NAC 5 mM) and with Povidon 5% (control) for 6 h. The percentages of viable (vital), apoptotic (apo) and necrotic cells (necr) were determined by FACS-analysis for Annexin V-FITC and Propidiumiodide. Values are means ± SEM of 4 (HT29, Chang Liver, AsPC-1 and BxPC-3) and 12 (HT1080) independent experiments with consecutive passages. Asterisk symbols on brackets indicate differences between treatment groups. *** p ≤ 0.001, ** p ≤ 0.01, * p ≤ 0.05 (one-way ANOVA). [file 1756-9966-29-21-S2.JPEG]

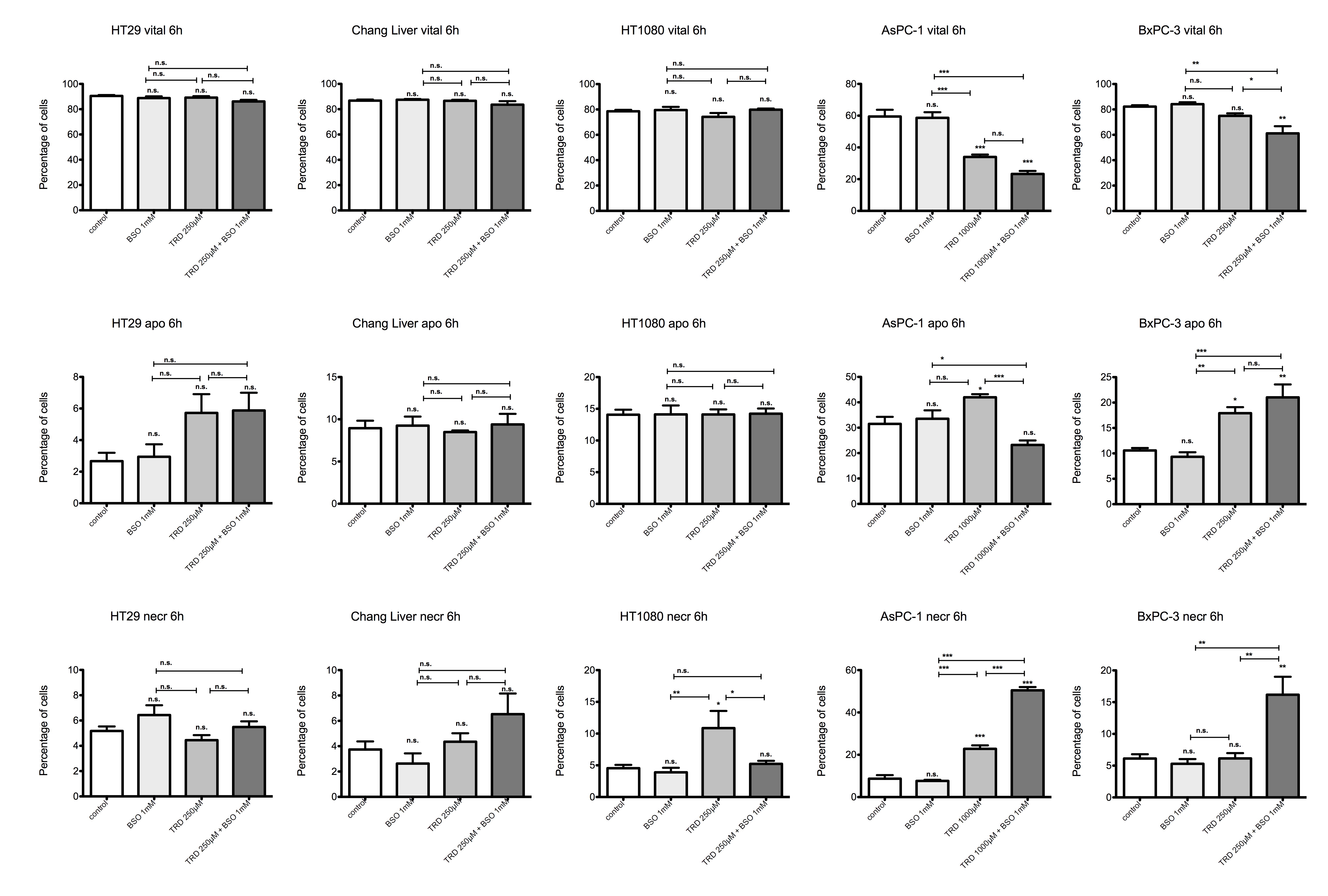

Supplement: Additional file 3 — Effects of DL-buthionin-(S,R)-sulfoximine on Taurolidine induced cell death in HT29, Chang Liver, HT1080, AsPC-1 and BxPC-3 cells after 6 h. HT29, Chang Liver, HT1080, AsPC-1 and BxPC-3 cells were incubated with either the glutathione depleting agent DL-buthionin-(S,R)-sulfoximine (BSO) (1 mM), Taurolidine (TRD) (250 μM) or the combination of both agents (TRD 250 μM + BSO 1 mM) and with Povidon 5% (control) for 6 h. The percentages of viable (vital), apoptotic (apo) and necrotic cells (necr) were determined by FACS-analysis for Annexin V-FITC and Propidiumiodide. Values are means ± SEM of 9 (HT29 and HT1080) and 4 (Chang Liver, AsPC-1 and BxPC-3) independent experiments with consecutive passages. Asterisk symbols on brackets indicate differences between treatment groups. *** p ≤ 0.001, ** p ≤ 0.01, * p ≤ 0.05 (one-way ANOVA). [file 1756-9966-29-21-S3.JPEG]
